# Crystal structure of 1,3,6,8-tetra­bromo-9-ethyl-9*H*-carbazole

**DOI:** 10.1107/S2056989015010117

**Published:** 2015-05-30

**Authors:** Mykola Bezuglyi, Gintare Grybauskaite, Gintautas Bagdziunas, Juozas Vidas Grazulevicius

**Affiliations:** a Department of Chemistry, National Taras Shevchenko University, 62a Volodymirska st., Kyiv, Ukraine; b Department of Polymer Chemistry and Technology, Kaunas University of Technology, Radvilenu Road 19, LT-50254, Kaunas, Lithuania

**Keywords:** crystal structure, carbazole, halogen–halogen contact

## Abstract

In the title compound, C_14_H_9_Br_4_N, the tricyclic ring system is almost planar (r.m.s. deviation for the 13 non-H atoms = 0.017 Å) and the methyl C atom deviates from the mean plane of the ring system by 1.072 (17) Å. In the crystal, Br⋯Br contacts [3.636 (3) and 3.660 (3) Å] slightly shorter than the van der Waals contact distance of 3.70 Å are seen.

## Related literature   

For applications of *N*-substituted carbazole derivatives in anti­cancer research, see: Caulfield *et al.* (2002 ▸). For their use in optoelectronic devices, see: Niu *et al.* (2011 ▸); Miyazaki *et al.* (2014 ▸); Grigalevicius *et al.* (2002 ▸).
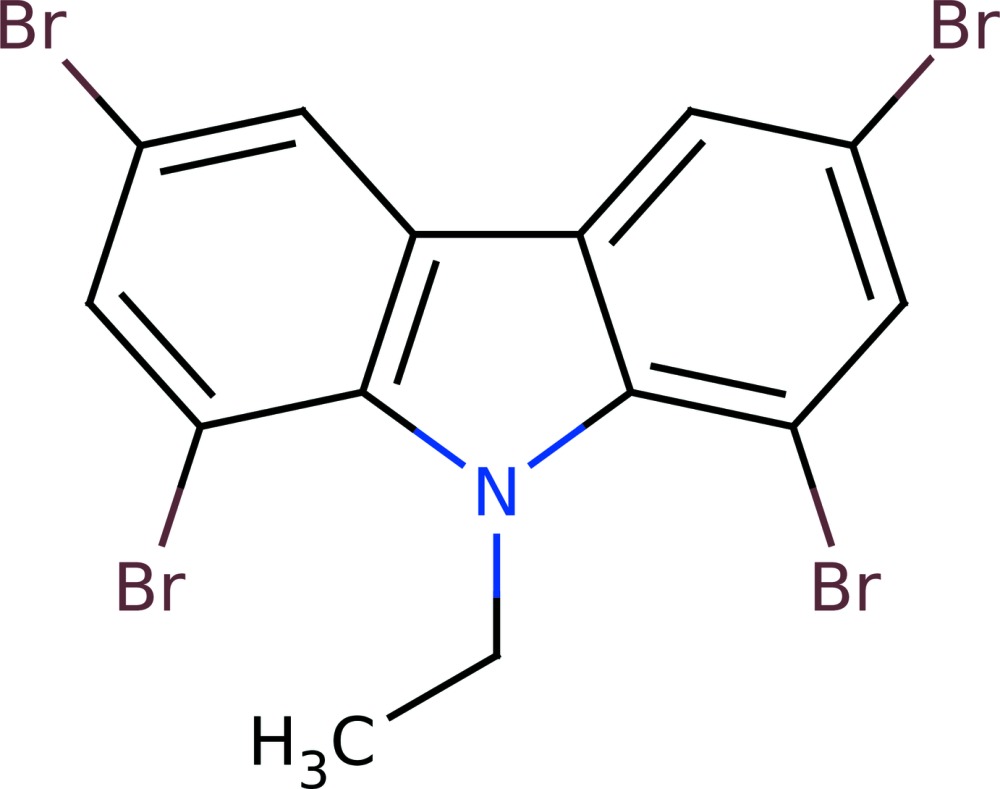


## Experimental   

### Crystal data  

C_14_H_9_Br_4_N
*M* *_r_* = 510.85
Monoclinic, 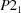

*a* = 4.202 (2) Å
*b* = 14.654 (6) Å
*c* = 12.245 (6) Å
β = 92.758 (18)°
*V* = 753.1 (6) Å^3^
*Z* = 2
Mo *K*α radiation
μ = 10.70 mm^−1^
*T* = 293 K
0.40 × 0.13 × 0.12 mm

### Data collection  

Rigaku XtaLAB mini diffractometer
Absorption correction: multi-scan (*REQAB*; Rigaku, 1998 ▸) *T*_min_ = 0.115, *T*_max_ = 0.277
2755 measured reflections
2599 independent reflections
2071 reflections with *F* ^2^ > 2.0σ(*F* ^2^)
*R*_int_ = 0.021

### Refinement  

*R*[*F* ^2^ > 2σ(*F* ^2^)] = 0.056
*wR*(*F* ^2^) = 0.130
*S* = 1.01
2600 reflections
172 parameters
1 restraint
H-atom parameters constrained
Δρ_max_ = 0.74 e Å^−3^
Δρ_min_ = −0.66 e Å^−3^
Absolute structure: Flack (1983 ▸), 868 Friedel Pairs
Absolute structure parameter: 0.05 (4)

### 

Data collection: *CrystalClear-SM Expert* (Rigaku, 2011 ▸); cell refinement: *CrystalClear-SM Expert*; data reduction: *CrystalClear-SM Expert*; program(s) used to solve structure: *SIR92* (Altomare *et al.*, 1994 ▸); program(s) used to refine structure: *SHELXL97* (Sheldrick, 2008 ▸); molecular graphics: *CrystalStructure* (Rigaku, 2010 ▸); software used to prepare material for publication: *CrystalStructure*.

## Supplementary Material

Crystal structure: contains datablock(s) General, I. DOI: 10.1107/S2056989015010117/hb7428sup1.cif

Structure factors: contains datablock(s) I. DOI: 10.1107/S2056989015010117/hb7428Isup2.hkl

Supporting information file. DOI: 10.1107/S2056989015010117/hb7428Isup3.cml

**Fig. 1.**
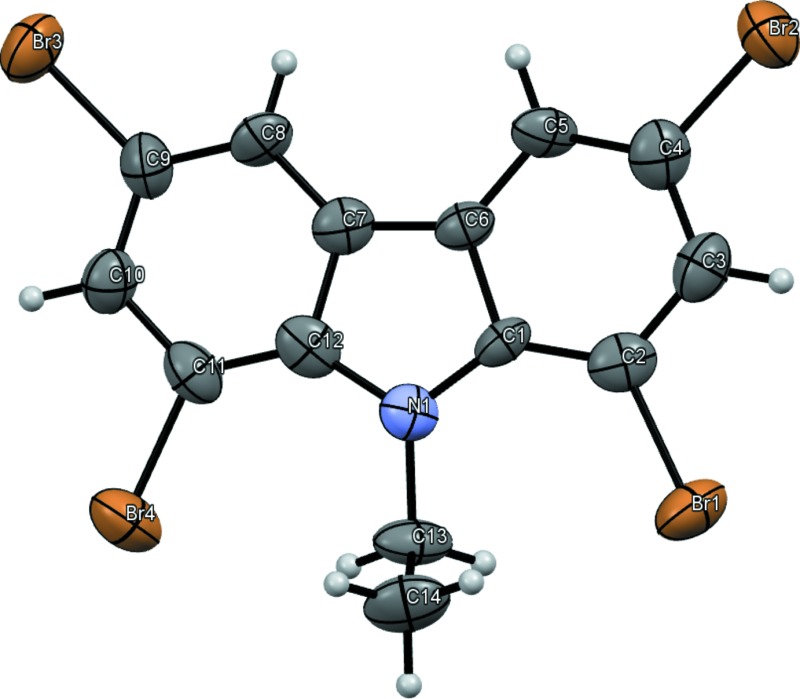
The molecular structure of the title molecule with displacement ellipsoids drawn at the 50% probability level.

. DOI: 10.1107/S2056989015010117/hb7428fig1.tif
The mol­ecular structure of the title mol­ecule with displacement ellipsoids drawn at the 50% probability level.

**Fig. 2.**
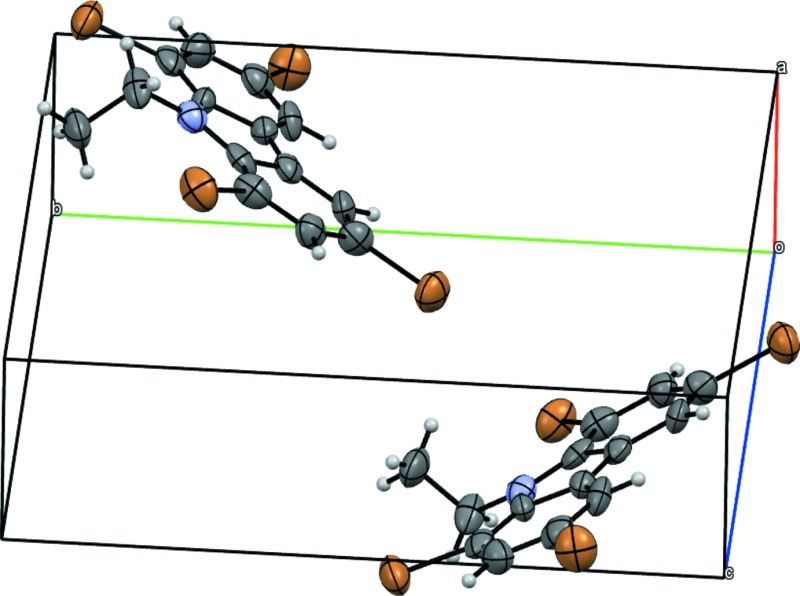
The crystal packing of the title compound.

. DOI: 10.1107/S2056989015010117/hb7428fig2.tif
The crystal packing of the title compound.

**Fig. 3.**
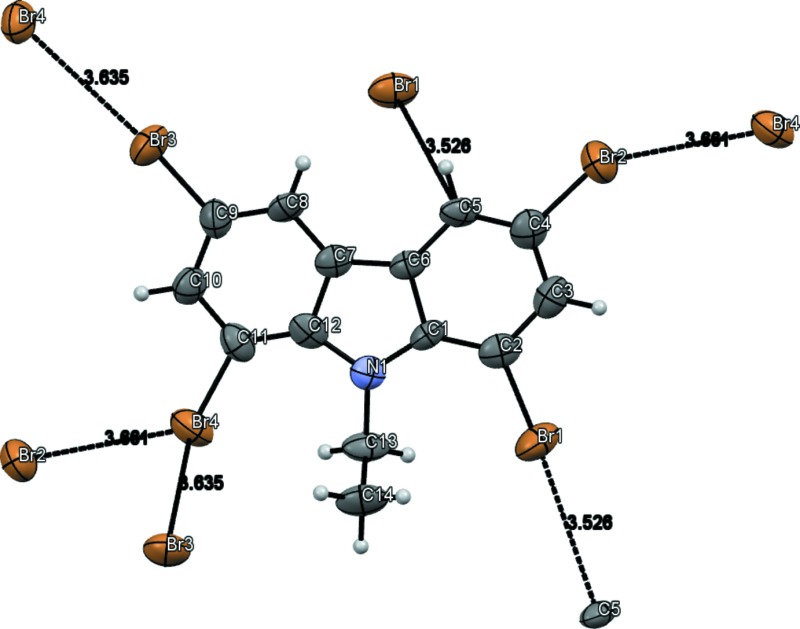
C—Br···Br and Br···π intermolecular contacts.

. DOI: 10.1107/S2056989015010117/hb7428fig3.tif
C—Br⋯Br and Br⋯π inter­molecular contacts.

CCDC reference: 1402621

Additional supporting information:  crystallographic information; 3D view; checkCIF report

## supplementary crystallographic information

### S1. Synthesis and crystallization

9-Ethyl-9*H*-carbazole (0.904 g, 4.63 mmol) was added to the solution of *N*-bromo­succinimide (NBS) (3.708 g, 20.83 mmol) in 30 ml of DMF. The reaction mixture was heated at 60°C for 24 hours. When the reaction completed (monitored *via* TLC) the solution was poured into a large amount of water with ice. The precipitate was filtered off and crystallized from the mixture of iso­propanol and DMF (volume ratio about 5:1) to isolate the product as needles. The bulk sample appears yellowish, but individual crystals are colourless. Yield 1.80 g (76 %), m.p. 155–156°C. ^1^H NMR (700 MHz, CDCl_3_) δ 7.92 (d, *J* = 1.8 Hz, 2H), 7.69 (d, *J* = 1.8 Hz, 2H), 5.10 (q, *J* = 7.1 Hz, 2H), 1.33 (t, *J* = 7.1 Hz, 3H).

### S2. Refinement

All hydrogen atoms were placed in geometrically idealized positions and constrained to ride on their parent atoms, with C—H = 0.930 Å for aromatic C—H, with 0.969 Å for methyl­ene C—H, 0.957 Å for methyl distances and U_iso_(H) = 1.2 U_eq_.

### Crystal data

**Table:**

C_14_H_9_Br_4_N	*F*(000) = 480.00
*M**_r_* = 510.85	*D*_x_ = 2.253 Mg m^−^^3^
Monoclinic, *P*2_1_	Mo *K*α radiation, λ = 0.71075 Å
Hall symbol: P 2yb	Cell parameters from 2306 reflections
*a* = 4.202 (2) Å	θ = 3.2–27.5°
*b* = 14.654 (6) Å	µ = 10.70 mm^−^^1^
*c* = 12.245 (6) Å	*T* = 293 K
β = 92.758 (18)°	Chip, colorless
*V* = 753.1 (6) Å^3^	0.40 × 0.13 × 0.12 mm
*Z* = 2	

### Data collection

**Table:**

Rigaku XtaLAB mini diffractometer	2071 reflections with *F*^2^ > 2.0σ(*F*^2^)
Detector resolution: 6.827 pixels mm^-1^	*R*_int_ = 0.021
ω scans	θ_max_ = 27.5°
Absorption correction: multi-scan (*REQAB*; Rigaku, 1998)	*h* = −5→5
*T*_min_ = 0.115, *T*_max_ = 0.277	*k* = −18→18
2755 measured reflections	*l* = −15→4
2599 independent reflections	

### Refinement

**Table:**

Refinement on *F*^2^	Hydrogen site location: inferred from neighbouring sites
*R*[*F*^2^ > 2σ(*F*^2^)] = 0.056	H-atom parameters constrained
*wR*(*F*^2^) = 0.130	*w* = 1/[σ^2^(*F*_o_^2^) + (0.0517*P*)^2^] where *P* = (*F*_o_^2^ + 2*F*_c_^2^)/3
*S* = 1.01	(Δ/σ)_max_ < 0.001
2600 reflections	Δρ_max_ = 0.74 e Å^−^^3^
172 parameters	Δρ_min_ = −0.66 e Å^−^^3^
1 restraint	Absolute structure: Flack (1983), 868 Friedel Pairs
Primary atom site location: structure-invariant direct methods	Absolute structure parameter: 0.05 (4)
Secondary atom site location: difference Fourier map	

### Special details

**Table:**

Refinement. Refinement was performed using all reflections. The weighted *R*-factor (*wR*) and goodness of fit (*S*) are based on *F*^2^. *R*-factor (gt) are based on *F*. The threshold expression of *F*^2^ > 2.0 σ(*F*^2^) is used only for calculating *R*-factor (gt).

### Fractional atomic coordinates and isotropic or equivalent isotropic displacement parameters (Å^2^)

**Table:**

	*x*	*y*	*z*	*U*_iso_*/*U*_eq_	
Br1	1.0896 (3)	0.54414 (9)	0.00371 (12)	0.0652 (4)	
Br2	0.4335 (4)	0.80930 (10)	−0.24666 (11)	0.0710 (4)	
Br3	0.3881 (3)	1.05176 (9)	0.38945 (12)	0.0657 (4)	
Br4	1.0717 (3)	0.73324 (10)	0.49105 (11)	0.0654 (4)	
N1	0.9560 (19)	0.7062 (6)	0.2062 (8)	0.041 (2)	
C1	0.854 (3)	0.7148 (7)	0.0995 (9)	0.037 (3)	
C2	0.893 (3)	0.6617 (8)	0.0045 (10)	0.046 (3)	
C3	0.768 (3)	0.6924 (8)	−0.0998 (10)	0.051 (3)	
C4	0.598 (3)	0.7757 (8)	−0.1069 (10)	0.048 (3)	
C5	0.556 (3)	0.8265 (7)	−0.0176 (9)	0.042 (3)	
C6	0.682 (3)	0.7978 (7)	0.0871 (9)	0.039 (3)	
C7	0.671 (3)	0.8383 (7)	0.1907 (9)	0.039 (3)	
C8	0.533 (3)	0.9191 (7)	0.2281 (10)	0.044 (3)	
C9	0.566 (3)	0.9430 (8)	0.3353 (9)	0.047 (3)	
C10	0.729 (3)	0.8854 (8)	0.4117 (10)	0.048 (3)	
C11	0.869 (3)	0.8032 (8)	0.3764 (9)	0.046 (3)	
C12	0.845 (3)	0.7785 (8)	0.2676 (9)	0.041 (3)	
C13	1.178 (3)	0.6333 (8)	0.2518 (11)	0.051 (3)	
C14	1.008 (3)	0.5530 (9)	0.2926 (12)	0.067 (4)	
H3	0.7995	0.6581	−0.1623	0.0616*	
H5	0.4442	0.8811	−0.0240	0.0498*	
H8	0.4181	0.9565	0.1791	0.0524*	
H10	0.7444	0.9015	0.4852	0.0581*	
H13A	1.3166	0.6139	0.1951	0.0616*	
H13B	1.3104	0.6590	0.3111	0.0616*	
H14A	0.8767	0.5270	0.2342	0.0802*	
H14B	0.8768	0.5712	0.3508	0.0802*	
H14C	1.1601	0.5084	0.3193	0.0802*	

### Atomic displacement parameters (Å^2^)

**Table:**

	*U*^11^	*U*^22^	*U*^33^	*U*^12^	*U*^13^	*U*^23^
Br1	0.0604 (7)	0.0431 (7)	0.0918 (11)	0.0099 (7)	0.0024 (7)	−0.0126 (7)
Br2	0.0871 (9)	0.0752 (10)	0.0491 (7)	0.0044 (9)	−0.0140 (7)	−0.0013 (7)
Br3	0.0759 (8)	0.0475 (7)	0.0738 (9)	0.0015 (8)	0.0042 (7)	−0.0187 (7)
Br4	0.0710 (9)	0.0678 (9)	0.0557 (8)	−0.0063 (8)	−0.0143 (7)	0.0171 (6)
N1	0.039 (5)	0.037 (5)	0.047 (5)	−0.009 (4)	−0.002 (4)	0.000 (4)
C1	0.034 (5)	0.026 (5)	0.051 (6)	0.003 (5)	0.004 (5)	−0.004 (5)
C2	0.031 (5)	0.040 (6)	0.066 (8)	−0.010 (5)	−0.001 (5)	0.003 (5)
C3	0.052 (6)	0.048 (7)	0.055 (7)	−0.009 (6)	0.007 (5)	−0.017 (6)
C4	0.050 (6)	0.040 (6)	0.054 (7)	−0.013 (6)	0.004 (6)	−0.006 (5)
C5	0.049 (6)	0.020 (5)	0.054 (7)	−0.003 (5)	−0.011 (5)	0.002 (4)
C6	0.037 (5)	0.033 (6)	0.046 (6)	−0.001 (5)	0.005 (4)	−0.002 (5)
C7	0.038 (5)	0.030 (5)	0.048 (6)	−0.017 (5)	0.002 (5)	0.002 (4)
C8	0.049 (6)	0.032 (6)	0.051 (7)	−0.007 (5)	0.001 (5)	0.002 (5)
C9	0.057 (7)	0.048 (6)	0.037 (6)	−0.015 (6)	0.002 (5)	−0.004 (5)
C10	0.054 (6)	0.042 (6)	0.049 (7)	−0.008 (6)	0.002 (6)	−0.007 (5)
C11	0.047 (6)	0.047 (7)	0.043 (6)	−0.012 (6)	−0.009 (5)	0.007 (5)
C12	0.036 (5)	0.041 (6)	0.047 (6)	−0.020 (5)	−0.002 (5)	0.004 (5)
C13	0.039 (6)	0.037 (6)	0.076 (9)	0.002 (5)	−0.019 (6)	0.007 (6)
C14	0.066 (7)	0.030 (6)	0.106 (11)	−0.004 (7)	0.013 (7)	0.018 (7)

### Geometric parameters (Å, º)

**Table:**

Br1—C2	1.912 (11)	C7—C12	1.458 (15)
Br2—C4	1.880 (12)	C8—C9	1.359 (16)
Br3—C9	1.894 (12)	C9—C10	1.412 (16)
Br4—C11	1.906 (11)	C10—C11	1.418 (16)
N1—C1	1.361 (14)	C11—C12	1.379 (15)
N1—C12	1.393 (14)	C13—C14	1.476 (17)
N1—C13	1.507 (14)	C3—H3	0.930
C1—C2	1.415 (16)	C5—H5	0.930
C1—C6	1.418 (14)	C8—H8	0.930
C2—C3	1.429 (17)	C10—H10	0.930
C3—C4	1.417 (16)	C13—H13A	0.970
C4—C5	1.340 (16)	C13—H13B	0.970
C5—C6	1.426 (15)	C14—H14A	0.960
C6—C7	1.404 (15)	C14—H14B	0.960
C7—C8	1.403 (15)	C14—H14C	0.960
			
Br2···Br4^i^	3.660 (3)	Br3···Br4^ii^	3.636 (3)
			
C1—N1—C12	110.4 (8)	Br4—C11—C12	125.5 (9)
C1—N1—C13	125.6 (9)	C10—C11—C12	120.3 (10)
C12—N1—C13	123.8 (9)	N1—C12—C7	106.2 (9)
N1—C1—C2	134.0 (9)	N1—C12—C11	135.2 (10)
N1—C1—C6	108.5 (9)	C7—C12—C11	118.5 (10)
C2—C1—C6	117.5 (10)	N1—C13—C14	113.0 (9)
Br1—C2—C1	124.5 (8)	C2—C3—H3	120.413
Br1—C2—C3	114.7 (9)	C4—C3—H3	120.407
C1—C2—C3	120.7 (10)	C4—C5—H5	119.576
C2—C3—C4	119.2 (11)	C6—C5—H5	119.568
Br2—C4—C3	116.3 (9)	C7—C8—H8	119.725
Br2—C4—C5	122.8 (9)	C9—C8—H8	119.729
C3—C4—C5	120.9 (11)	C9—C10—H10	119.964
C4—C5—C6	120.9 (10)	C11—C10—H10	119.962
C1—C6—C5	120.9 (10)	N1—C13—H13A	108.986
C1—C6—C7	107.8 (9)	N1—C13—H13B	108.988
C5—C6—C7	131.3 (9)	C14—C13—H13A	108.984
C6—C7—C8	133.2 (10)	C14—C13—H13B	108.981
C6—C7—C12	106.9 (9)	H13A—C13—H13B	107.779
C8—C7—C12	119.9 (10)	C13—C14—H14A	109.473
C7—C8—C9	120.5 (10)	C13—C14—H14B	109.466
Br3—C9—C8	122.1 (9)	C13—C14—H14C	109.472
Br3—C9—C10	117.2 (9)	H14A—C14—H14B	109.470
C8—C9—C10	120.6 (11)	H14A—C14—H14C	109.470
C9—C10—C11	120.1 (11)	H14B—C14—H14C	109.476
Br4—C11—C10	114.2 (8)		
			
C1—N1—C12—C7	−2.6 (10)	C3—C4—C5—C6	−0.3 (16)
C1—N1—C12—C11	178.7 (10)	C4—C5—C6—C1	−0.2 (15)
C12—N1—C1—C2	−179.2 (9)	C4—C5—C6—C7	−179.9 (9)
C12—N1—C1—C6	3.2 (10)	C1—C6—C7—C8	−179.3 (9)
C1—N1—C13—C14	−92.7 (12)	C1—C6—C7—C12	0.9 (10)
C13—N1—C1—C2	4.9 (16)	C5—C6—C7—C8	0.4 (18)
C13—N1—C1—C6	−172.6 (8)	C5—C6—C7—C12	−179.4 (9)
C12—N1—C13—C14	91.9 (11)	C6—C7—C8—C9	−178.4 (10)
C13—N1—C12—C7	173.4 (8)	C6—C7—C12—N1	1.0 (10)
C13—N1—C12—C11	−5.3 (17)	C6—C7—C12—C11	179.9 (8)
N1—C1—C2—Br1	6.9 (16)	C8—C7—C12—N1	−178.9 (8)
N1—C1—C2—C3	−176.1 (9)	C8—C7—C12—C11	0.1 (14)
N1—C1—C6—C5	177.7 (8)	C12—C7—C8—C9	1.4 (15)
N1—C1—C6—C7	−2.5 (10)	C7—C8—C9—Br3	179.4 (8)
C2—C1—C6—C5	−0.3 (13)	C7—C8—C9—C10	−2.4 (16)
C2—C1—C6—C7	179.4 (8)	Br3—C9—C10—C11	−179.7 (7)
C6—C1—C2—Br1	−175.7 (7)	C8—C9—C10—C11	2.0 (16)
C6—C1—C2—C3	1.3 (13)	C9—C10—C11—Br4	−178.9 (9)
Br1—C2—C3—C4	175.5 (7)	C9—C10—C11—C12	−0.5 (16)
C1—C2—C3—C4	−1.7 (15)	Br4—C11—C12—N1	−3.7 (17)
C2—C3—C4—Br2	−178.4 (8)	Br4—C11—C12—C7	177.7 (6)
C2—C3—C4—C5	1.2 (16)	C10—C11—C12—N1	178.1 (10)
Br2—C4—C5—C6	179.4 (6)	C10—C11—C12—C7	−0.5 (15)

Symmetry codes: (i) *x*−1, *y*, *z*−1; (ii) −*x*+1, *y*+1/2, −*z*+1.
