# Supplementary figures and images for: Crystal structure of 1,3,6,8-tetra­bromo-9-ethyl-9H-carbazole
Source: Acta Crystallogr E Crystallogr Commun. 2015 May 30;71(Pt 6):o373. doi: 10.1107/S2056989015010117 (PMC4459356; doi:10.1107/S2056989015010117)

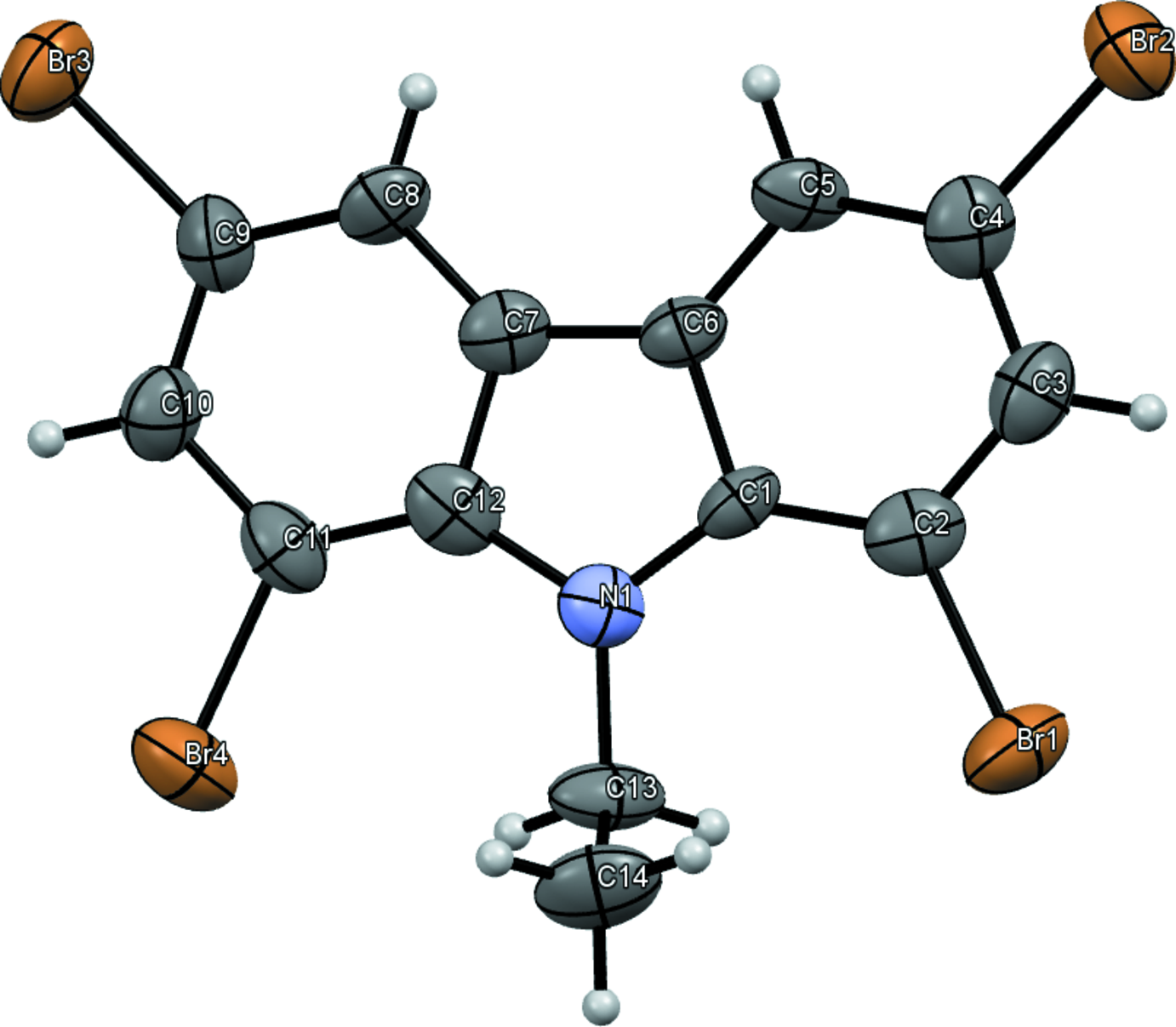

Supplement: Supplementary file 4 [file e-71-0o373-fig1.tif]

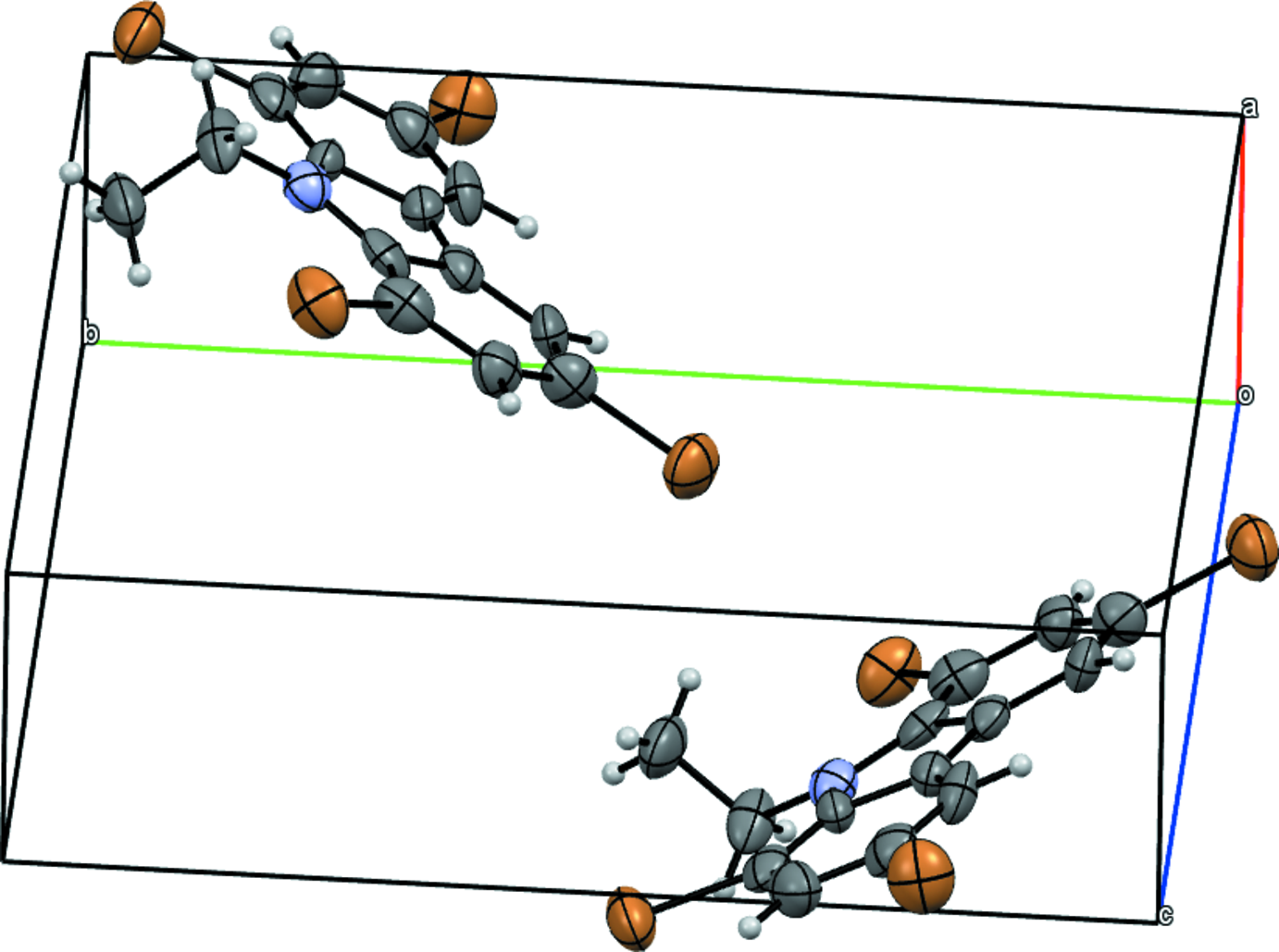

Supplement: Supplementary file 5 [file e-71-0o373-fig2.tif]

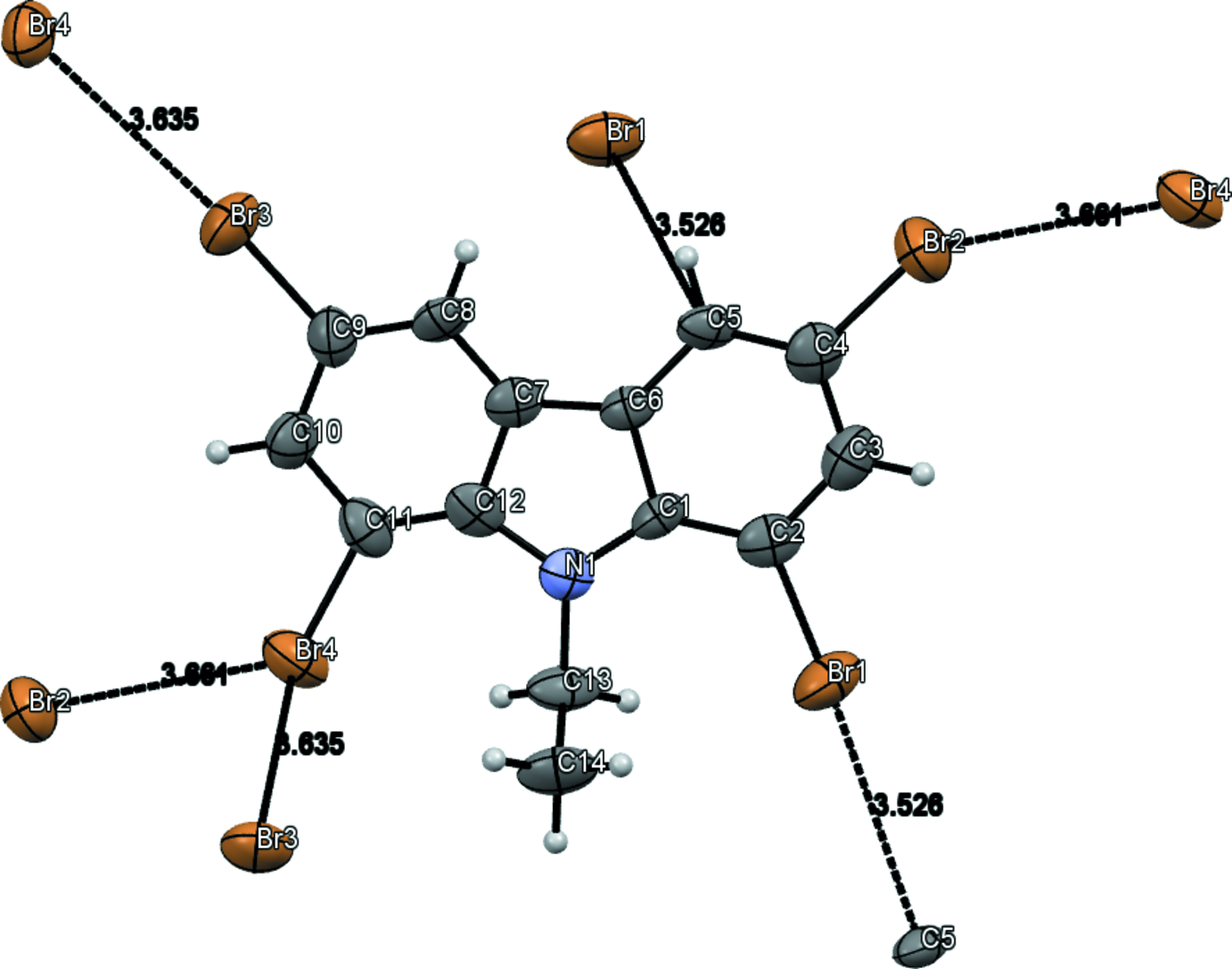

Supplement: Supplementary file 6 [file e-71-0o373-fig3.tif]
